# Screening for Tumor-Specific Antibodies by Yeast Display of Native Repertoires of Tumor-Infiltrating B Cells

**DOI:** 10.32607/actanaturae.27909

**Published:** 2026

**Authors:** Y. R. Vereveyko, D. M. Malabuiok, E. A. Faizullina, Yu. A. Mokrushina, O. V. Serova, S. V. Protsenko, N. Gong, H. Zheng, Yu. B. Kuzmin, P. V. Tsarapaev, I. V. Smirnov, N. E. Kushlinskii, H. Zhang, A. G. Gabibov, S. S. Terekhov

**Affiliations:** Shemyakin–Ovchinnikov Institute of Bioorganic Chemistry of the Russian Academy of Sciences, Moscow, 117997 Russia; State Key Laboratory of Medicinal Chemical Biology and College of Life Sciences, Nankai University, Tianjin, 300350 China; N.N. Blokhin National Medical Research Center of Oncology of the Russian Ministry of Health, Moscow, 115522 Russia; Endocrinology Research Center, Ministry of Health of the Russian Federation, Moscow, 117036 Russia; Faculty of Chemistry, Lomonosov Moscow State University, Moscow, 119991 Russia

**Keywords:** tumor-infiltrating B cells (TIBs), tumor antigens (TAs), yeast display, biopanning, microfluidics, reconstruction of native B cell repertoires, tumor-specific antibodies

## Abstract

Tumors are neoplasms that can be recognized by a patient’s immune system. Recognition of tumor antigens (TAs) triggers the recruitment and proliferation of tumor-infiltrating lymphocytes. Although tumor-infiltrating T cells have long been the primary focus of research, B cells also accumulate in tumors and participate actively in TA recognition. The use of molecular display technologies for the functional profiling of tumor-infiltrating B cell (TIB) repertoires enables large-scale screening for TAs and identification of unique TIB clones that mediate selective tumor recognition. Approaches combining microfluidic technologies for reconstructing natural TIB repertoires with yeast display were used in this study to identify antibodies that recognize TAs. It has been shown that nonspecific adhesion of yeast cells to the surface of primary tumor cell cultures from patients may be a key factor hindering the isolation of tumor-specific clones. Overcoming nonspecific binding of yeast cells enables the selection of tumor-specific clones by biopanning. Clone 3.15, isolated from the TIB repertoire of a colorectal cancer (CRC) patient, recognizes TAs on the surface of a number of tumor cell lines, as well as on primary tumor cell cultures from CRC patients. We suggest that the further use of these technologies will not only expand the diversity of molecular tools for targeted immunotherapy and tumor diagnostics but will also provide a more detailed understanding of the molecular mechanisms underlying the generation and recognition of TAs.

## INTRODUCTION

Cancer ranks second among the causes of death in Russia. Worldwide, approximately 20 million new cancer cases and 9.7 million cancer deaths are reported annually [1]. The advent of immunotherapy has revolutionized oncology, significantly improving patient survival and reshaping the treatment landscape for a number of malignancies [2]. However, the range of molecular targets currently used in targeted therapy remains limited [3], requiring the identification of novel targets for the development of effective therapeutic agents.

Historically, research into the recognition of tumor-specific antigens has focused primarily on cytotoxic T cells as the key cells mediating antitumor immunity. Adoptive cell therapy, targeted antibody therapy, and treatment with immune checkpoint inhibitors, in particular, have directed the patient’s own T cell-mediated antitumor immunity against the tumor [4]. Unlike the native functional role of cytotoxic T cells, B cells can assume a dual function in tumor pathogenesis [5]. This dual role of B cells was for a long time a decisive factor in the choice of T cells as the primary focus of research on tumor-infiltrating lymphocytes. However, breakthroughs in understanding the functional role of tumor-infiltrating immune cells have revealed that tumor-infiltrating B cells (TIBs), and the population of antibody-producing plasma cells in particular, are of fundamental importance for a favorable prognosis [6]. The current concept posits that TIBs play a critical role in tumor control by ensuring the synergistic effect of B- and T-cell immunity [7]. Tumor-infiltrating lymphocytes frequently form tertiary lymphoid structures where B-cell activation, proliferation, and maturation take place, thus playing a pivotal role in shaping the antitumor immune response [8]. Therefore, research into the functional role of TIB repertoires allows for both elucidating new mechanisms of tumor pathophysiology and identifying novel molecular targets for developing targeted immunotherapeutics.

Antibodies in TIB repertoires can target not only neoantigens arising from somatic mutations in tumor cells, but also non-self proteins from oncogenic viruses [9], which may act as targets for cancer immunotherapy [10]. Even more importantly, native human proteins adopting abnormal conformations upon malignant transformation can act as tumor antigens [11]. Therefore, there is a virtually inexhaustible diversity of molecular determinants that are selective for the tumor microenvironment.

Previously employed approaches to the identification of molecular interactions between antibodies and antigens in tumors have been based on low-throughput methods enabling the functional analysis of only the most abundant clones of tumor-infiltrating B cells [9, 10]. The application of molecular display technologies to screen for abnormal antigen–antibody interaction interfaces enables more efficient screening for novel immunotherapy targets [11]. The use of microfluidic technologies and synthetic biology allows for the transformation of B-cell repertoires into highly representative yeast cell libraries, while preserving the native combinations of immunoglobulin heavy- and light-chain genes [12]. In addition, the molecular display technologies [13] enable the large-scale functional profiling of B cells. Therefore, it becomes feasible to identify both the most prevalent antibodies and the minor clones that are often the most biologically significant [14].

In this study, microfluidic technologies were employed to construct native TIB repertoires. Highly representative cell libraries were generated by cloning natively paired variable fragments of antibody heavy (VH) and light (VL) chains into an expression vector, followed by the transformation of *Pichia pastoris* yeast cells. For selection, the antibodies were directly screened from yeast libraries (biopanning) against antigenic determinants on the surface of tumor cell monolayers. We observed pronounced nonspecific binding of yeast cells to certain cell lines and primary cultures of patient-derived tumor cells during biopanning for tumor-specific clones. Therefore, the optimization of biopanning conditions was a critical step for the efficient selection of tumor-specific TIB clones, enabling the identification of the tumor-specific clone 3.15. The isolation of clone 3.15 paves the way for a detailed characterization of the molecular mechanisms underlying tumor recognition and the development of an immunotherapeutic agent based on this antibody.

## EXPERIMENTAL

**Cell cultures**

Primary cultures of cells isolated from the tumors of six colorectal cancer (CRC) patients who had undergone examination and treatment at the N.N. Blokhin National Medical Research Center of Oncology of the Russian Ministry of Health, were included in the study. All study procedures complied with the ethical standards of the Institutional Ethics Committee and the 1964 Declaration of Helsinki with its subsequent amendments, or equivalent ethical norms. Written informed consent was obtained from each participant enrolled in the study. In all patients, the clinical diagnosis was verified by a histomorphological examination of the tumor according to the WHO Classification of Tumours of the Digestive System (2019). The HEK-293T, HCT116, BT-474, MDA-MB-231, A431, and MDA-MB-468 cell lines were purchased from the American Type Culture Collection (ATCC).

**Tumor cell isolation**

Primary tumor cell cultures were isolated in accordance with the procedure described earlier [15]. A tumor fragment measuring ~ 0.5–2 cm^3^ was placed into a tube with phosphate-buffered saline (PBS) immediately after resection. The specimen was pelleted in a benchtop centrifuge (Eppendorf, Germany) for 5 min (100 *g,* 24°C), minced with a scalpel, and treated with an enzyme cocktail: 0.5 mg of DNase (Sigma, USA), 1 mg each of collagenase type I and type IV (Merck, USA), and 2 mg of hyaluronidase (Microgen, Russia) in a working medium (a 1 : 1 mixture of DMEM/F12 and RPMI 1640 (PanEco, Russia), supplemented with 10% fetal bovine serum (FBS; HyClone, Cytiva, USA), 2 mM *L*-alanyl-*L*-glutamine (Yeasen, China), and a 1× antibiotic–antimycotic solution (Gibco, USA)). The tube was placed on a rotary platform (Biosan, Latvia) and incubated for 40 min at 7 rpm in a CO_2_ incubator at 37°C in an atmosphere of 8% CO_2_. After the incubation, the cell suspension was pipetted to dissociate aggregates and passed sequentially through cell strainers with pore sizes of 100, 70, and 40 µm. The cells were pelleted by centrifugation for 15 min (300 *g* at 24°C). The resulting cell pellet was resuspended in 1 mL of ACK buffer (150 mM ammonium chloride, 10 mM potassium bicarbonate, and 0.1 mM EDTA) for 1 min to achieve erythrocyte lysis, and the reaction was terminated by adding 2 mL of the working medium. The cells were then pelleted by centrifugation (7 min, 300 *g* at 24°C), and the pellet was resuspended in 2 mL of a culture medium composed of 90% Advanced DMEM (Thermo Fisher Scientific, USA), 10% FBS (HyClone, Cytiva), 2 mM *L*-alanyl-*L*-glutamine, and an antibiotic–antimycotic mixture (levofloxacin 1 µg/mL, polymyxin B 20 µg/mL, vancomycin 20 µg/mL, meropenem 20 µg/mL, micafungin 10 µg/mL, and voriconazole 10 µg/mL). The resulting cells were cultured until colony formation and cloned using a cloning cylinder. The resulting primary tumor cell cultures were tested for sterility and mycoplasma contamination.

**Reconstruction of native B-cell repertoires**

The cells derived from the tumor specimens were encapsulated in water-in-oil emulsion droplets using a microfluidic chip with flow-focusing geometry (nozzle diameter, 60 µm; hydrophobic Aquapel coating) [16]. In the first channel, cells were supplied at a concentration corresponding to an average droplet occupancy of λ = 0.1 for TIBs (~5 × 10^6^ cells/mL). TIBs concentration was determined by flow cytometry. In the second channel, which was connected to the first one immediately prior to droplet formation, a suspension of oligo-dT microspheres (NEB, USA) in lysis buffer (100 mM Tris-HCl, pH 7.5; 500 mM LiCl; 10 mM EDTA; 5 mM DTT; 1% LiDS) was supplied. The resulting mixture was combined with two outer channels containing Pico-Surf 2 fluorocarbon oil (Dolomite, UK) using the flow-focusing geometry to yield a microfluidic water-in-oil emulsion. Cell lysis and hybridization of cellular mRNA to oligonucleotides immobilized on the microsphere surface took place within the emulsion. Following emulsion breakage, the purified microspheres were re-encapsulated to conduct emulsion reverse transcription-PCR (eRT-PCR) [17]. The *VH* and *VL* genes were amplified using a qScript One-Step RT-qPCR Kit (Quantabio, USA) and a panel of degenerate primers complementary to the leader sequences (forward primers L-VH1-4 for) and J segments (reverse primers VHJ1-6 rev) of the heavy chain genes, as well as to the variable segments (forward primers VK1-7 for and VL1-5 for) and the beginning of the C segment (reverse primers Ck rev and CL rev) of the kappa and lambda light chain genes. The physical binding of the VH and VL chains into a single amplicon was mediated by overlapping 4GS linker sequences encoded within the forward VK/VL and reverse VHJ primers. The next stage involved nested PCR to enhance amplification specificity and insert the universal SfiI restriction sites for subsequent amplicon cloning into a vector for the expression of single-chain variable fragments (scFv) anchored to the surface of *Pichia pastoris* yeast cells.

**Generation of the cell libraries of natively paired scFv**

A yeast vector based on the pPIC9K plasmid was used to generate natively paired scFv libraries in *Pichia pastoris*. The vector contains an inducible alcohol oxidase (*AOX1*) promoter that drives *scFv* gene expression after adding methanol as the sole carbon source to the growth medium, as well as the modified signal peptide sequence of *Saccharomyces cerevisiae* α-factor (α-short), the 3xFLAG epitope tag, the *SAG1* α-agglutinin gene for antibody presentation on the yeast cell surface, and the auxotrophic *HIS4* marker for transformant selection [16]. The VH–VL amplicons were cloned into the SfiI restriction sites, followed by electroporation into highly competent *Escherichia coli* DH10B cells. The resulting libraries contained approximately 1 × 10^6^ individual clones. The scFv libraries in the pPIC9K_anch vector were isolated from bacterial biomass using a QIAGEN Plasmid Midi Kit according to the manufacturer’s protocol. The resulting plasmid DNA was linearized at the PmeI restriction site and used to transform the histidine-auxotrophic *P. pastoris* GS115 strain. Electrocompetent *P. pastoris* cells were prepared by treatment with lithium acetate supplemented with dithiothreitol [17]. Selection was performed using a minimal RBD agar medium without histidine added.

**Molecular display and the biopanning procedure**

The library of yeast transformants was grown in YPD liquid medium at an initial optical density of OD_600_ = 0.1 for 24 h (30°C, 220–250 rpm). To induce the expression of anchored scFv, the cells were transferred to BMMY medium (20 g/L peptone, 10 g/L yeast extract, 13.4 g/L YNB with ammonium sulfate, 0.4 mg/L biotin, 100 mM potassium phosphate buffer, pH 6.0, and 0.5% methanol) at an initial OD_600_ = 2 and cultured for 2 days (30°C, 220 rpm). To maintain a high scFv expression level, methanol was added every 24 h to a final concentration of 0.5%. On the second day of incubation in the expression medium, the cells were pelleted by centrifugation (1,500 *g*, 5 min) and used for screening. For the selection of tumor-specific clones, a tumor cell culture at 100% confluence was washed with PBSCMA (1× PBS containing 1 mM CaCl_2_, 0.5 mM MgCl_2_, and 0.5% BSA) supplemented with blocking agents to minimize nonspecific yeast binding (0.1% casein for optimal blocking). Yeast cells were added at a density of OD_600_ = 5 for the first selection round and OD_600_ = 0.5 for the subsequent rounds (10 mL per 90 cm^2^ Petri dish) and incubated for 1 h at room temperature. The nonadherent yeast cells were collected and discarded; the nonspecifically bound yeast cells were washed thrice with PBSCMA supplemented with 0.1% casein. Yeast clones binding to the tumor cell culture were evaluated visually using an optical microscope. The tumor cell culture and associated yeast cells were detached from the Petri dish by adding a trypsin–EDTA solution. The resulting cell suspension was plated onto YPD agar medium supplemented with 100 µg/mL ampicillin and 100 µg/mL kanamycin, followed by a 48-h incubation at 30°C for yeast clone regeneration. Following the incubation, the yeast cell pool was recovered from the plates using liquid YPD medium; the optical density was measured at 600 nm. Some cells were used for further selection, while the remainder was frozen in YPD medium supplemented with 15% glycerol using liquid nitrogen and stored at −80°C. Four sequential enrichment rounds for the yeast clones displaying tumor-specific scFv on their surface were conducted. Clone representation was assessed on the MGI platform using VH amplicons generated via ePCR of the corresponding regions of genomic DNA from the yeast pools [18].

**Reconstruction of full-length antibodies**

Antibody heavy chain sequences were cloned into the universal pFUSE-CHIg-hG1 vector at the EcoRI/BsmBI restriction sites; the light chain sequences were cloned into either the pFUSE2-CLIg-hK or pFUSE2-CLIg-hL2 depending on the isotype of the light chain of the selected clones (κ or λ) at the EcoRI/BbsI or EcoRI/AvrII restriction sites, respectively. The *VH* and *VL* gene sequences were amplified using genomic DNA from the selected clones as a template. The fidelity of assembly of the expression constructs was verified by Sanger sequencing. The resulting constructs were used for transient transfection of HEK-293F cells. Cells were transfected using polyethyleneimine (PEI, 25K; Polysciences, USA) to produce recombinant full-length antibodies. The cells were cultured in FreeStyle 293 medium in an incubator at 125 rpm and 37°C in an atmosphere of 5% CO_2_ for 6 days. The cell suspension was centrifuged; the supernatant was filtered through a 0.2 µm membrane and used for IgG1 purification by two-step chromatographic procedures. The first purification step involved affinity chromatography on a HiTrap Protein G HP column (Cytiva). Size-exclusion chromatography on a Superdex 200 10/300 GL column (Cytiva) was conducted as the second step. The purity of the antibody preparations was analyzed by sodium dodecyl sulfate-polyacrylamide gel electrophoresis followed by densitometry.

**Flow cytometry**

Cells were dissociated using a 0.25% trypsin-EDTA solution. Cell counting and viability assessment were performed on a CellDrop FL cell counter (DeNovix, USA); 10 µL of the 1 : 1 mixture of the cell suspension and trypan blue dye were applied. The cells were stained and washed in buffer PBE containing 1× PBS (pH 7.4), 0.5 % BSA, and 2 mM EDTA. The cells were pelleted by centrifugation at 300 *g* for 5 min and washed with PBE to remove residual culture medium. The cells were then incubated with primary antibodies at a concentration of 3 µg/mL in 100 µL of PBE for 30 min. Following incubation, the cells were washed twice with five volumes of PBE and resuspended in 100 µL of PBE containing secondary anti-human IgG-DL650 antibodies (Thermo Fisher Scientific) at a 1 : 500 dilution, followed by a 30-min incubation. SYTOX Blue dye (Thermo Fisher Scientific) was added to the cells 10 min before the end of the incubation with antibodies to exclude dead cells according to the manufacturer’s instructions. The cells were then washed twice with five volumes of PBE, resuspended in 100 µL of the same buffer, and analyzed on a NovoCyte flow cytometer (ACEA Biosciences, USA).

## RESULTS AND DISCUSSION

**The application of molecular display technologies for functional screening of TIB repertoires**

An approach based on biopanning as a critical step in isolating tumor-specific clones from TIB repertoires was proposed as the key strategy for identifying tumor-specific antibodies (*Fig. 1*).

**Fig. 1:**
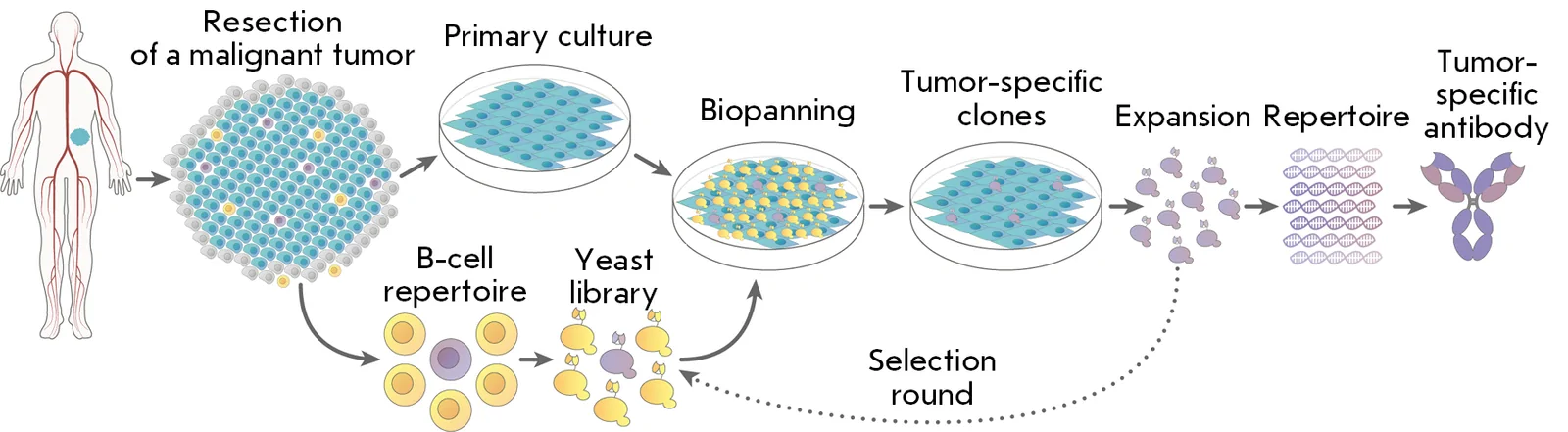
Schematic representation of the identification of tumor-specific antibody clones from TIB repertoires

Immediately after tumor resection, a surgical specimen was subjected to dissociation to obtain a primary tumor cell culture. Variable fragments of IgG antibodies were simultaneously reconstructed from patient’s tumor-infiltrating B cells as a *P. pastoris* yeast scFv library. Microfluidic technologies made it possible to encapsulate individual TIBs in microfluidic emulsion droplets and preserve the native combinations of the VH and VL domains of the antibodies. The resulting yeast library carrying the scFv of the patient’s native antibody repertoire was used for biopanning. An autologous primary tumor cell monolayer was utilized as a substrate to select tumor-specific clones. The yeast library suspension was incubated with the primary tumor cell culture. The yeast clones that did not bind to the primary cell culture monolayer were removed by washing, yielding a subpopulation of tumor-specific yeast clones exhibiting increased affinity for tumor cells. These clones were used for subsequent selection rounds. In most cases, three or four selection rounds were sufficient to statistically increase the titer of tumor-specific clones. Subpopulations of tumor-specific clones were sequenced via high-throughput sequencing, which made it possible to assess the enrichment of individual clones of tumor-specific antibodies. The identified antibodies were reconstructed as full-length IgG1 immunoglobulins, and their binding to tumor cells was validated.

**Overcoming the nonspecific binding of yeast cells to tumor cells**

Selective binding of tumor-specific clones is one of the key prerequisites for their effective enrichment through biopanning. Meanwhile, we found that certain cell lines (such as HCT116) and primary tumor cultures from CRC patients exhibited extremely high levels of nonspecific binding to *P. pastoris* cells *(Fig. 2*). We utilized various agents to mitigate nonspecific binding, including the conventional blocking agents used in enzyme-linked immunosorbent assay (BSA and casein), sucrose, biocompatible block copolymer surfactants (Kolliphor P188 and Pluronic F127), and inactivated *P. pastoris* cells (*Fig. 2*).

**Fig. 2:**
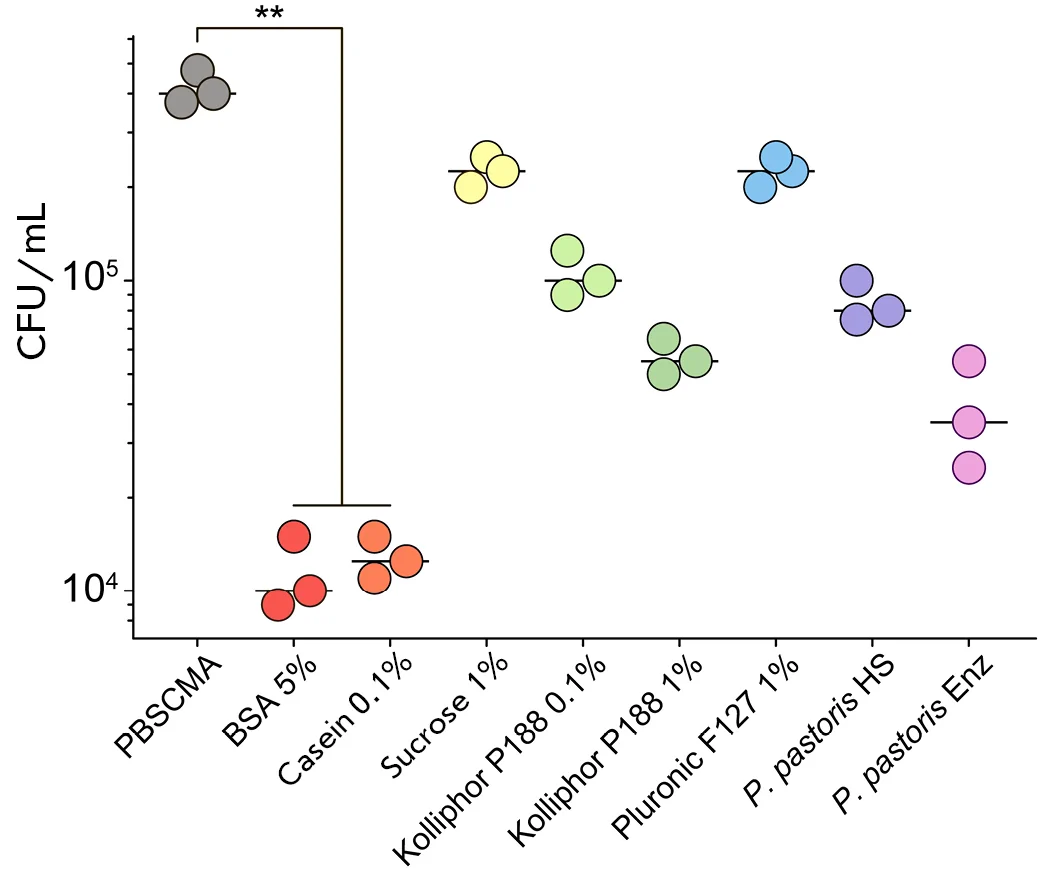
Effect of agents blocking nonspecific binding of yeast cells to tumor cells from a primary culture of a CRC patient. The buffer PBSCMA (1× PBS containing 0.5 mM MgCl_2_, 1 mM CaCl_2_, and 0.5% BSA) for biopanning was supplemented with various blocking agents, including *P. pastoris* cells inactivated by heat shock (HS) or the lytic enzyme zymolyase (Enz). The titer of *P. pastoris* bound to tumor cells was determined in three independent biological replicates. *P*-values were calculated using the *t*-test, **P < 0.01

The addition of 5% BSA or 0.1% casein to the basal buffer PBSCMA statistically significantly (*P* < 0.01, *t*-test) reduced the level of nonspecific binding by more than 1.5 orders of magnitude. The other components, primarily Kolliphor P188 and inactivated *P. pastoris* cells, also reduced nonspecific binding, although less efficiently. Therefore, the use of 5% BSA or 0.1% casein was considered optimal.

To test whether the targeted binding of tumor-specific clones was preserved, we evaluated the effect of the selected blocking agents on a model system: *P. pastoris* cells expressing the anchored scFv of the trastuzumab antibody (4D5) [19]. 4D5 highly efficiently binds the tumor-associated antigen HER2, which is overexpressed in certain cancers. Cells expressing the scFv of the D16 antibody, which binds the RBD domain of the SARS-CoV-2 S protein, were used as a negative control. The HER2+ breast cancer cell line BT-474 was used as a model substrate to assess the binding efficiency of the tumor-specific clone (4D5) and the nonspecific binding of the irrelevant clone (D16). Blocking agents were shown to have no impact on the selection efficiency of tumor-specific clones, as they statistically significantly (*P* < 0.01, *t*-test) reduced the nonspecific binding of *P. pastoris* D16 cells to BT-474 cancer cells (*Fig. 3*).

**Fig. 3:**
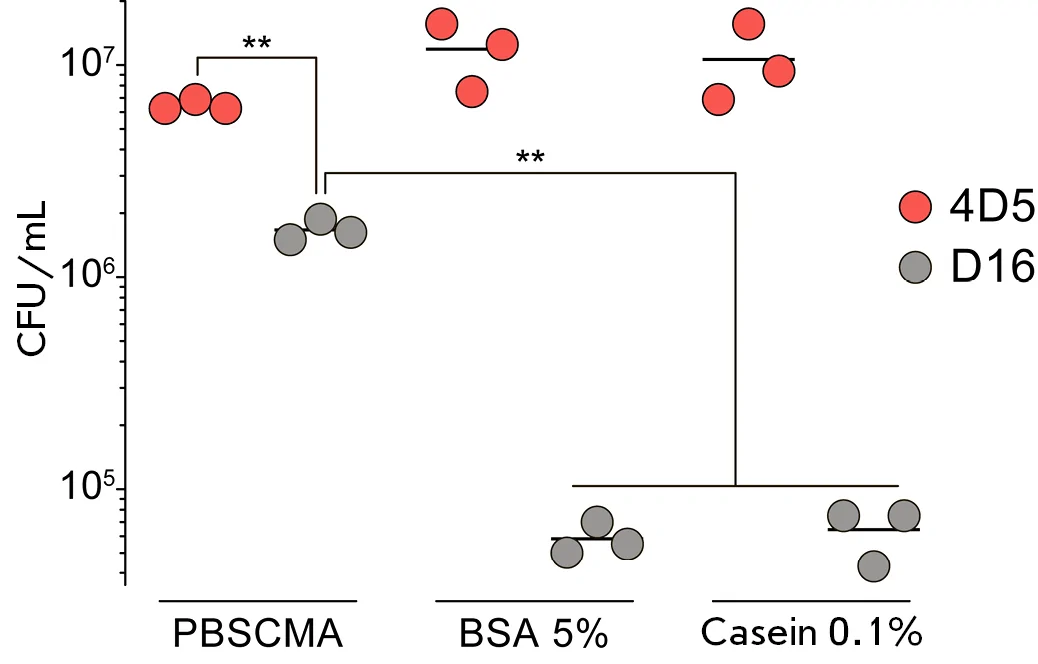
Effect of blocking agents on specific binding to the model HER2-positive cell line BT-474. 4D5 indicates the model tumor-specific antibody (trastuzumab); D16 indicates the irrelevant control antibody. The titer of *P. pastoris* bound to tumor cells was determined in three independent biological replicates. *P*-values were calculated using the *t*-test, **P < 0.01

In the model library containing 1% of tumor-specific 4D5 *P. pastoris* cells, PBSCMA used without any blocking agents resulted in limited enrichment after a single selection round (2 ± 0.3-fold), which was insufficient for the efficient selection of tumor-specific clones (*Fig. 4*).

**Fig. 4:**
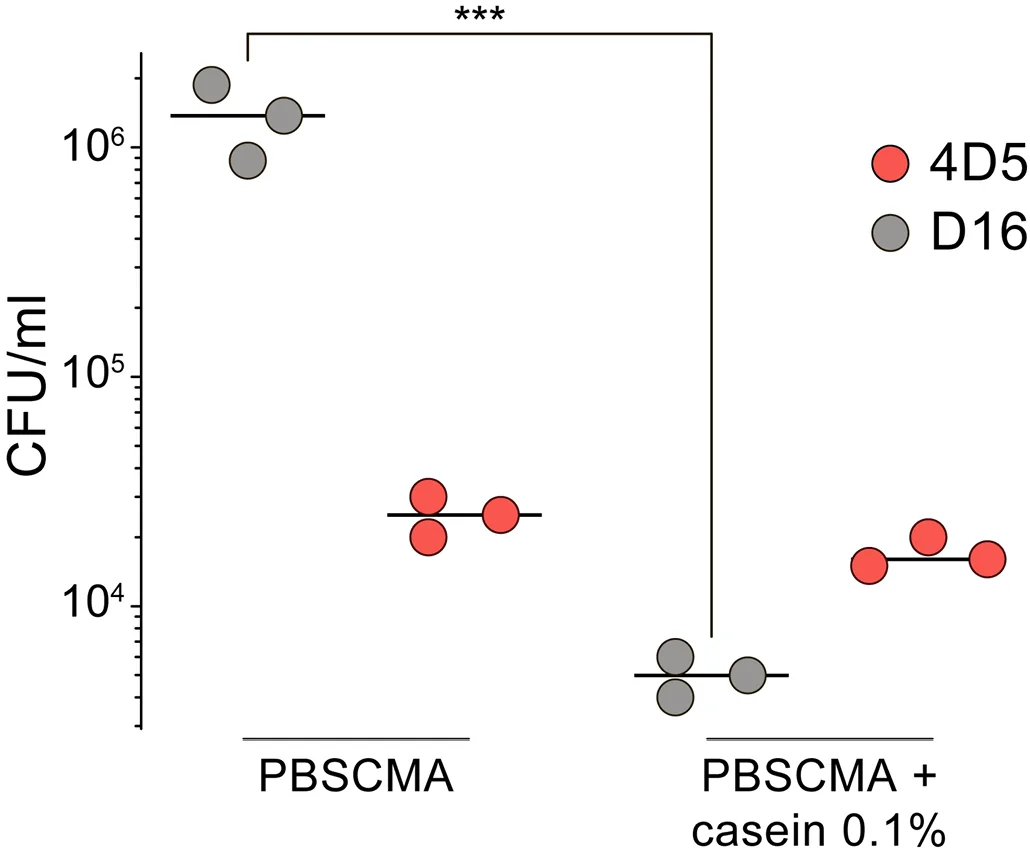
Efficiency of selecting tumor-specific clones from the model yeast library containing 1% tumor-specific clones (4D5) in the presence of an excess of irrelevant control clones (D16). The HER2-positive cell line BT-474 was used as the model tumor cells. The titer of *P. pastoris* bound to tumor cells was determined in three independent biological replicates. *P*-values were calculated using the *t*-test, ***P < 0.001

The addition of 0.1% casein reduced the proportion of irrelevant control *P. pastoris* (D16) cells in the mixture after selection by two orders of magnitude, indicating that the pool of tumor-specific clones was significantly enriched (77 ± 12-fold). The resulting selection efficiency enables the isolation of even minor tumor-specific clones (< 0.001%) within three selection rounds.

**Selection of the tumor-specific antibody clone 3.15 from the TIB repertoire of the CRC patient**

We used optimized selection conditions to screen for tumor-specific clones from the TIB repertoire of the CRC patient. The library was constructed using approximately 10^5^ TIBs; the estimated representativeness of the yeast library was 10^6^ clones, making it possible to efficiently represent the TIB diversity in the analyzed patient’s specimen.

Three biopanning rounds were performed to select the tumor-specific clone 3.15; statistically significant enrichment of the library with tumor-specific clones was observed as early as during the second round. High-throughput sequencing allowed us to monitor the abundance of clone 3.15 according to the sequence of the third CDR region in the heavy chain of the antibody (HCDR3) during selection (*Fig. 5*).

**Fig. 5:**
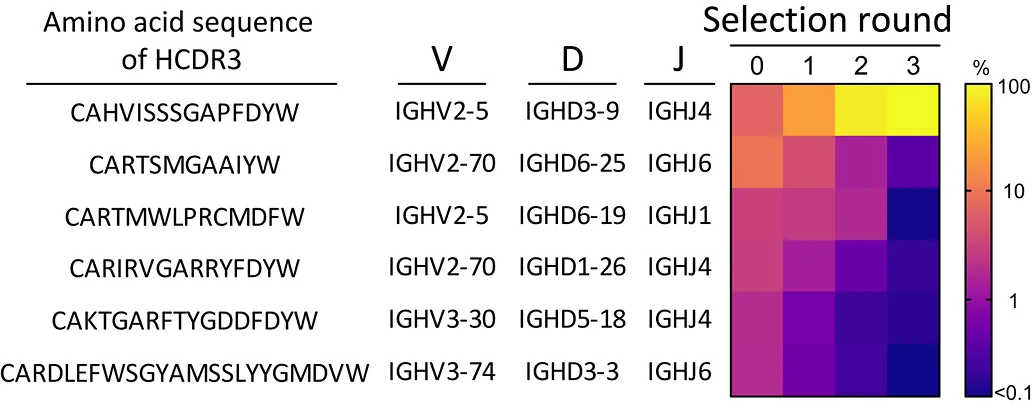
Distribution of the most prevalent clones in the CRC patient library during selection. The frequency of the individual clones in the library is presented as a heatmap; the color scale indicates the percentage of clones with a specific HCDR3 sequence in each selection round

Clone 3.15 was not minor, constituting approximately 4% of the population prior to selection, which may indicate its active function in the tumor. After three selection rounds, clone 3.15 constituted more than 90% of the tumor-specific clone subpopulation (*Fig. 5*).

In order to validate its binding to tumor cells, clone 3.15 was reconstructed as a full-length IgG1 antibody. The antibody clone 3.15 efficiently bound to the primary cell culture derived from the patient (*Fig. 6A*), as well as to the BT-474 cell line and, to a lesser extent, to MDA-MB-231, HEK-293T, and A431 cells, without binding to MDA-MB-468 cells (*Fig. 6B*).

**Fig. 6:**
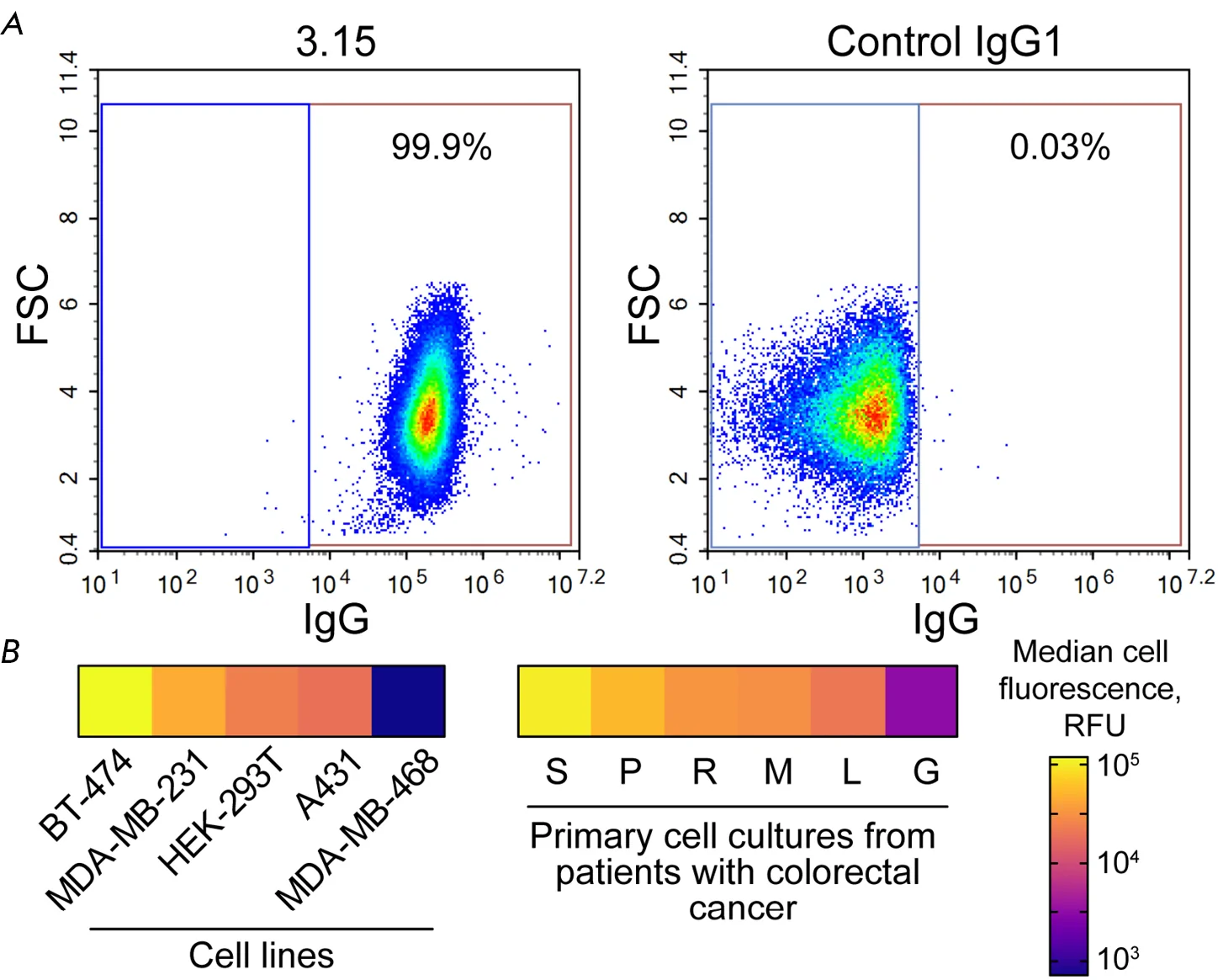
Binding of antibody 3.15 to tumor cell lines and primary cell cultures from CRC patients. (*A*) Flow cytometry of the autologous primary culture from the CRC patient S. Tumor cells were incubated with antibody 3.15 or an isotype control antibody (IgG1, clone D16) and then stained with secondary antibodies conjugated to the far-red fluorophore DyLight 650. (*B*) Median fluorescence of cells from various tumor cell lines and primary cell cultures from CRC patients (denoted as S, P, R, M, L, G), presented as a heatmap. The color scale indicates the fluorescence of the major subpopulation of living cells, expressed in relative fluorescence units (RFU)

Moreover, the antibody clone 3.15 bound to five out of six primary cell cultures from CRC patients. Our findings provide a proof of concept that the developed approaches can be applied to effectively identify tumor-specific antibodies. Further research into the specificity of the antibody clone 3.15 is highly relevant for identifying novel molecular targets for cancer immunotherapy.

## CONCLUSIONS

Despite the tremendous success of targeted immunotherapeutic approaches based on antibodies and their derivatives (antibody–drug conjugates (ADCs), bispecific antibodies (BsAbs), and chimeric antigen receptors (CARs)), the range of molecular targets remains limited. Most antibodies that are currently in use were generated via hybridoma technologies. However, methods based on functional profiling of individual cells can reshape the existing market for monoclonal antibody drugs. An analysis of patients’ B-cell repertoires allows for the direct identification of functional variants of human antibodies, as well as the discovery of novel molecular targets.

The biopanning technology based on yeast display methods has been optimized in this study to perform screening of tumor-specific clones from native TIB repertoires. The detailed analysis of TIB repertoires is of exceptional importance, as it enables direct identification of human monoclonal antibodies recognizing tumor antigens. The antibody clone 3.15 is an example showing how these technologies can be implemented to develop novel immunotherapeutics based on immunoglobulins from cancer patients. Our findings indicate that the antibody clone 3.15 is not patient-specific; therefore, detailed characterization of its antigenic specificity is highly relevant for future research. The results of this study demonstrate that tumors from five out of six enrolled CRC patients can potentially be targeted using immunotherapeutics based on the antibody clone 3.15 or its optimized variants. These preliminary results provide a basis for suggesting that the antibody clone 3.15 holds promise for the targeted therapy of colorectal cancer, which requires further validation.

The approaches developed in this study are of high practical significance, since they allow for the efficient identification of novel tumor-specific antibodies. Furthermore, a detailed analysis of the antigenic specificity of antibodies from TIB repertoires will make it possible to identify novel molecular targets. We believe that the antibodies identified from TIB repertoires will increase the selectivity of immunotherapeutics, since the recognition of novel molecular tumor determinants that are not typical of healthy human tissues underlies their selection. A detailed structural analysis of the molecular targets of antibodies from TIB repertoires will shed light on how the immune system recognizes tumor antigens, thereby opening new avenues for developing novel immunotherapeutic approaches with enhanced efficacy and selectivity.
